# A Spectral Method for Rapidly Determining the Linear Birefringence of Thin Polymer Films

**DOI:** 10.3390/molecules29246007

**Published:** 2024-12-20

**Authors:** Dana Ortansa Dorohoi, Dan Gheorghe Dimitriu

**Affiliations:** Faculty of Physics, Alexandru Ioan Cuza University, 11 Carol I Blvd, RO-700506 Iasi, Romania; dimitriu@uaic.ro

**Keywords:** channeled spectra, linear birefringence, PVA and PET thin stretched films

## Abstract

A rapid and simple spectral method for determining the linear birefringence of thin anisotropic films, using the channeled spectra, is proposed in this article. Two channeled spectra must be recorded for a transparent system containing a thick anisotropic layer and a thin stretched polymer film, when the two anisotropic uniaxial layers have parallel and perpendicular optical axes, respectively. The sum and difference of the two channeled spectra indicate (by the positions of the maxima and minima in the resulting channeled spectra) the phase difference introduced by the thin polymer film. One must measure with precision only the thickness of the polymer film in order to compute the linear birefringence of the thin polymer film, using the position of the maxima and minima of the sum and difference. The experimental data obtained for poly (vinyl alcohol)—PVA—and poly (ethylene terephthalate)—PET—films attest to the applicability of the proposed method to the uniaxial transparent polymeric thin films.

## 1. Introduction

The method described in this manuscript is devoted to determining small values of the optical pathways introduced by anisotropic uniaxial thin layers between the ordinary and extraordinary components of linearly polarized light in the propagation process. The optical pathway is determined by both the thickness and birefringence of the anisotropic layer. The experiment described here asks for thin uniaxial films, because the linear birefringence of the stretched polymer films, or that of the liquid crystalline layers [1,2] is higher than that of the inorganic crystals [3,4].

Polymers are a category of materials with a wide area of applications due to their physical properties.

Each polymer layer shows an intrinsic optical anisotropy [1] caused by the asymmetry of the molecular structure and also by the random disorder of its side-chains. The polymer anisotropy can be increased by external actions (mechanical, electric, or magnetic fields). Among the various procedures for increasing the total anisotropy of polymer layers [1,5,6], the most used is stretching under gentle heating. The induced anisotropy, achieved by polymer film stretching, is added to the intrinsic one. The total polymer anisotropy is dependent on the molecular structure, on the initial thickness of the layer subjected to external forces, and also on the light spectral composition. The linear birefringence characterizing the anisotropic polymer layers must be determined when they are used in devices working in total polarized light.

The stretching direction of the transparent polymer layer becomes its optical axis. The optical axis and two perpendicular axes (named principal axes) form a triangular system of coordinates in which the matrix of the refractive indices has only diagonal elements differing from zero. The linearly polarized light does not change its polarization state in the propagation process parallel to the principal axes. The light velocity does not depend on its polarization state when light propagates along the optical axis.

The linear birefringence, Δ*n*, is a material-dependent parameter defined as the difference between the extraordinary and ordinary refractive indices of a uniaxial anisotropic material [7]. It depends on the light wavelengths, being a dispersive parameter, due to the dependence of both refractive indices on the light spectral composition:

(1) $$\Delta n=n_{e}-n_{o}$$

In relation (1), indices *e* and *o* refer to the extraordinary and ordinary refractive indices of the material measured with linearly polarized light having the electric field parallel and perpendicular, respectively, to the optical axis of the anisotropic material.

The linear birefringence can be determined by different methods. Such methods can be based on Brewster’s law, Snell’s law of refraction, or on the formula of the critical angle [8]. The second series of methods is based on interferometric measurements [9,10,11,12]: they can offer a direct estimation of the birefringence [1,10] with high precision.

When the thick polymer layers are not transparent enough, or one needs thin polymer films with known birefringence, one uses special methods for measurements. Special compensators are able to offer information about the value of the birefringence, but the measurements are made with monochromatic radiations and cannot offer information about the birefringence dispersion.

A special interferometric method, previously proposed, consists of recording the channeled spectrum of the anisotropic layer introduced between two polarizing filters [1,11]. The recorded channels are null if the two polarizing filters are used with their transmission directions either parallel or crossed. This method is applicable to all transparent uniaxial anisotropic layers [1,3,10].

The device, consisting of the anisotropic layer between the crossed polarizers, can also be used for determining the flux densities corresponding to the small and high axes of the light polarizing ellipse in order to estimate the phase difference introduced between the electric fields acting on the principal axes of the anisotropic layer [13]. This method must be used only for very thin anisotropic layers due to the periodicity of trigonometric function cosine, which does not allow precision in the evaluation of the optical phase difference. In order to avoid non-determination due to the periodicity of trigonometric function cosine, the method based on the polarizing ellipse [13] can be used with two identical anisotropic layers having comparable thicknesses and mounted with their optical axes perpendicular to each another. In this way, one decreases the optical pathway introduced by the anisotropic sample, because the thickness of the anisotropic layer determining the optical pathway is equal to the difference of the thicknesses of the two layers.

The linear birefringence of the stretched polymer layers is usually higher than that of the inorganic crystalline layers, but the very thin films introduce small a optical pathway between the ordinary and extraordinary components of linearly polarized light that cannot be emphasized by interferometric methods.

The previous method proposed by us [10,11] for the optical characterization of the thin film polymer anisotropy supposes measurements of the channel’s position in two types of channeled spectra obtained by using a thick anisotropic layer and a stretched thin polymer film, when they have parallel and perpendicular optical axes, respectively. The two types of channeled spectra offer information both for the film’s anisotropy and its dispersion, as was demonstrated.

In the new procedure, described in this article, the sum and the difference of the channeled spectra (recorded in the precedent step of the experiment) are computed. From the channeled spectra representing the sum and the difference of the channeled spectra recorded with the two anisotropic layers having their optical axes perpendicular and then parallel to each another, one can estimate, with enough precision, the linear birefringence of the polymer film, if the thickness of the thin layer is known or can be measured with enough precision. This new method can be applied both for polymers and for very thin crystalline layers, or for very thin liquid crystal films.

This new method has been introduced because the method based on the polarization ellipse is not applicable, due to the periodicity of function cosines [13], and the method using the compensators asks for measurements for each wavelength, for which the birefringence of the film is unknown, and also does not offer enough precision [1].

Due to relatively high values of the polymer birefringence, measurements must be made with thin layers so as to not exceed the coherence length of light when it becomes non-polarized [7]. In this case, the thin polymer anisotropic layers show a small number of channels in the visible range, and the measurements using the channeled spectra cannot be made.

The phase difference introduced by the thin layer is small indeed, but when it is added or subtracted from the phase difference introduced by a thick anisotropic layer, it becomes measurable. In this case, the new method based on the sum and difference of the channels, respectively, has the advantage that the positions of the maxima and minima are easily observed.

Due to the multiple and various applications of PVA and PET polymeric films [14,15], especially when they work in totally polarized light, as well as to the multitude of studies regarding their linear birefringence [1,5,6,9,10,11,12,13], the thin films of poly (vinyl alcohol), PVA, and poly (ethylene terephthalate), PET [1,10], were chosen to demonstrate the applicability of the new method proposed here for determining the polymer linear birefringence. The transparent thin layers from PVA and PET were stretched, and their linear birefringence determined by the new method was compared with that estimated by other methods.

PVA is a granular polymer insoluble in cold water as well as a great number of organic solvents. It is soluble in hot water, allowing one to obtain, by boiling, homogeneous solutions which can be dried and then stretched. Finally, one can obtain transparent, flexible, and elastic films. The linear birefringence of the stretched PVA films depends on both the thickness of the initially unstretched film and its degree of stretching [1,13]. Polymer anisotropy can be modified by mechanical actions on the polymer layer before complete drying (rubbing with a velvet textile, scratching with sandpaper of low roughness, or processing with wire brush) [1], especially for preparing the supports for liquid crystalline layers [16] used for the increasing of the linear birefringence of devices working in total polarized light.

The transparent PVA layers are used as supports for liquid-crystal displays (LCDs) or for organic light-emitting diodes (OLEDs) [16,17,18].

High performing displays ask for high transparency, birefringence, and dichroism of the polymer films in order to ensure a high contrast ratio, large viewing angle, color conservation, and so on [18].

In medicine, PVA films are used in ophthalmology for contact lens fabrication. Due to their biodegradability, PVA films are also used in drug delivery and tissue replacement for improving or correcting human organs [19,20,21,22].

There is a wide area of applications of PVA layers in industry.

The optical devices contain polarizing filters from PVA working by dichroism, or they are used as optical compensators.

Due to the flexibility of the polymer chains, PVA exhibits durability, high transparency, covalent cross-linking ability, chemical stability, pH and thermal stability, and easy and low costs process ability; it is therefore commonly introduced in the composition of the electro-active polymers.

PVA films containing various stick molecules become dichroic by stretching; their dichroism depends on the degree of orientation and helps spectroscopists to establish the directions of the charge displacement in electronic transitions caused by the interaction with light [23,24,25,26].

The PET films were also considered as a possible material for proving the validity of the proposed method because they were studied from an optical point of view by other methods and there are existing experimental data about their birefringence [10,13].

PET is a semi-crystalline or amorphous thermoplastic polymer with a partially aliphatic and aromatic structure which can be drawn on to improve its crystallinity through increasing the reciprocal orientation of its molecular chains [27,28], thereby increasing its anisotropy.

Like PVA, PET has low fabrication costs and is a recyclable material [28,29,30]. PET is the most recyclable plastic to date, and it has high chemical resistance to acids, bases, and solvents; it is also a barrier for moisture, gases, and other environmental factors.

In contact with living tissues, PET does not introduce any negative reactions, being a known biocompatible material. It can be used for medical implants such as coatings, cardiovascular prostheses [31], and blood vessels’ metallic stents, having the ability to reduce thrombus formation [32,33,34].

PET is used in dentistry for improving cell biocompatibility, increasing cell production and adhesion, and enhancing osteo-integration [35,36]. Due to its resistance to dye staining, PET is adequate as a dental material.

In the PET process of fabrication, the polymeric chains mostly lie in-plane, and a supplementary uniaxial tension applied to the sample has a slight impact on the orientation of the molecular chains situated in the out-of-plane direction [37,38,39]. So, when unidirectional stress is applied, the in-plane parameters can be drastically modified, while the out-of-plane parameters remain unchanged [37,38]. Due to the very close values of the two extraordinary refractive indices corresponding to the out-of-plane polymeric chains, differing from the value measured for the in-plane refractive index (ordinary refractive index), PET can be considered with enough precision as a uniaxial polymer with its optical axis parallel to the stretching direction [39,40,41].

The anisotropy of PET films determines their usage in obtaining polarizing filters, LCD displays, or optical fibers.

The anisotropic properties of PVA and PET films can be modified in the manufacturing processes to obtain the asked values for the linear birefringence required for each optical device.

Due to the dependence of the linear birefringence of the polymer layers on both initial thickness and stretching degree, each stretched polymer film must be analyzed by optical means before its usage in making devices working in polarized light.

## 2. Experimental Setup

**Figure 1:**
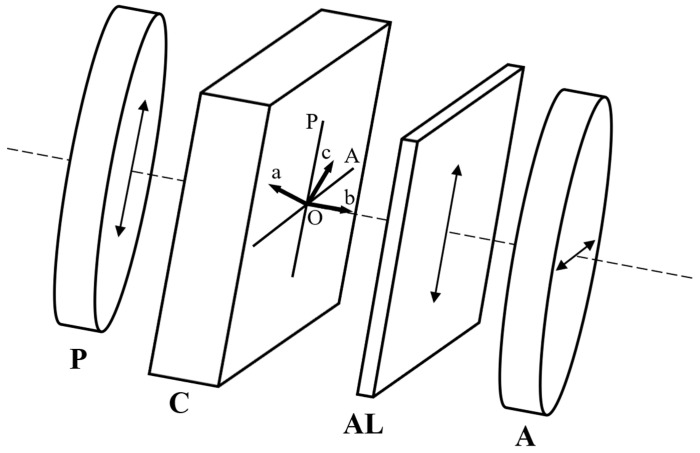
Device D for channeled spectra recording. P and A, identical polarizing filters with crossed transmission directions; C, thick crystalline layer; AL, anisotropic thin layer. The arrows drawn on the polarizers P and A represent their transmission directions, while the arrow drawn on the anisotropic layer AL represents its optical axis.

The experimental device D (see Figure 1) used for determining the channeled spectra consists of two identical polarizing filters, P and A, which are mounted with either parallel or perpendicular transmission directions, having between them the anisotropic layer C. The device D is placed in the measure beam of a spectrophotometer with two beams. Two identical polarizers with their transmission directions in parallel are introduced in the compensatory beam for compensatory reasons. One thin uniaxial layer, AL, can be placed in D between the thick crystalline layer and the analyzer A.

**Figure 2:**
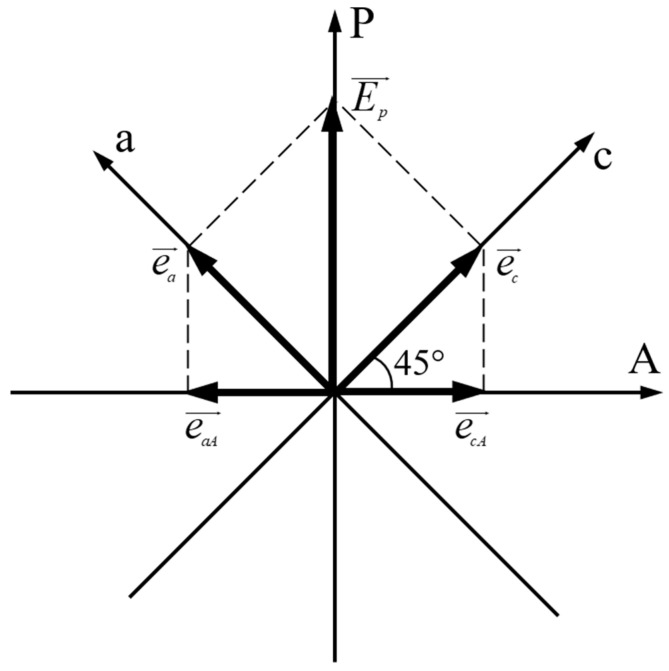
Relative orientation of the transmission directions of polarizers P and A and the main axes O*a* and O*c* of the anisotropic layers. Light propagates along O*b*.

In our laboratory, the channeled spectra were recorded with a Specord UV Vis Carl Zeiss Jena spectrophotometer with a data acquisition system. The device D was used with crossed transmission directions and the angle between the main axes of C and the transmission directions of the polarizers was 45 degrees (Figure 2).

**Figure 3:**
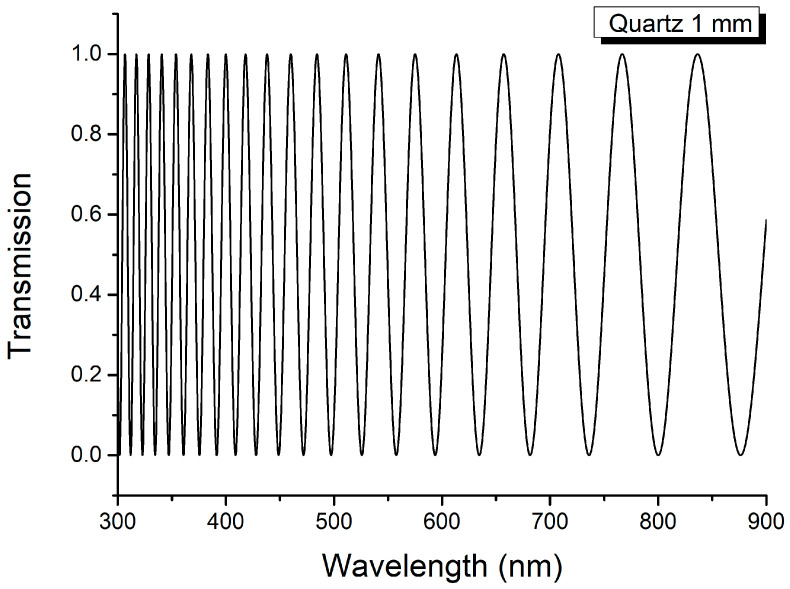
Channeled spectrum of crystalline layer C (*L*_1_ = 1 mm = 10^−3^ m, Δ*n* = 0.0092).

To apply the method proposed here for measuring the linear birefringence of thin anisotropic layers, the channeled spectrum of the thick uniaxial crystal C (in our experiment, a quartz crystal with Δ*n*_1_ = 0.0092 and *L*_1_ = 1 mm = 10^−3^ m) is initially recorded; it has channels in the visible range (see Figure 3).

Two other channeled spectra are recorded when the polymer film is introduced in the device D from the measure beam: the first when the optical axes of the two anisotropic layers are parallel and the second when their optical axes are perpendicular.

The linear birefringence of the thin polymer layer is then estimated using the sum and the difference of the channeled spectra recorded with the crystal and the film (having optical axes in parallel and perpendicular to each another).

## 3. Theoretical Notions

The transmission factor *T*(*λ*) of the device D (introduced in the measure beam of the spectrophotometer) is defined [7,11] as the ratio between the linearly polarized flux emerging from the second polarizing filter A, *ϕ_e_*(*λ*), and the incident flux, *ϕ_i_*(*λ*), on the polarizing filter P in Figure 1:

(2) $$T\left(\lambda \right)=\frac{\phi_{e}\left(\lambda \right)}{\phi_{i}\left(\lambda \right)}$$

The incident flux on the entrance surface of the crystal C is $\frac{1}{2}\phi_{i}$, because the polarizing filter works by dichroism. If one supposes that light propagates along the O*b* axis of crystalline layer C, the components of the electric field of wave on the principal axes O*a* and O*c* of crystal can be written as follows:

(3) $$e_{a}=E_{P}\cos\alpha \cos\left(\omega t+\psi_{0}\right)$$

(4) $$e_{c}=E_{P}\sin\alpha \cos\left(\omega t+\psi_{0}+\Delta \psi_{ac}\right)$$

In Equations (3) and (4), e and E denote the elongation and the amplitude of the electric field of light, respectively, and the indices *a*, *c*, and *p* denote the projections of the light electric field on the principal axes O*a* and O*c* and also on the polarizing filter P. *ω* is the wave pulsation, *t*-time, *ψ*_0_ is the initial phase of the wave at the entrance in the crystal C, and Δ*ψ_ac_* is the phase difference between the components *e_a_* and *e_c_* at the exit from the crystalline layer. The angle α between the transmission direction of P and the optical axis O*c* of crystalline layer C is usually 45 degrees in order to ensure the equality of the flux densities of ordinary and extraordinary light components. This value of angle α ensures the null channels in the recorded spectrum after device D.

When the crystal C is placed between the polarizing filters, the phase difference between the light components *e_a_* and *e_c_* depends on the linear birefringence Δ*n*_1_, on the thickness *L*_1_ of crystal C, and on the wavelength *λ* of incident light:

(5) $$\Delta \psi_{ac}=\frac{2\pi }{\lambda }\Delta n_{1}L_{1}$$

The phase difference between the ordinary and extraordinary components of light was introduced only in one equation, when relations (3) and (4) were written.

Using the formula for the light flux [11] at the exit of the crystal, with $\Delta \psi_{ac}=\Delta \psi_{1}$, one can write the light flux after the analyzer A:

(6) $$\phi_{e}=\frac{\phi_{i}}{2}\left(1\pm \cos\Delta \psi_{1}\right)$$

The sign + or − in (6) shows that the polarizing filters have their transmission directions parallel or orthogonal, respectively. According to relation (2), the transmission factor of the system D (illustrated in Figure 1) is as follows:

(7) $$T\left(\lambda \right)=\frac{1}{2}\left(1\pm \cos\Delta \psi_{1}\right)$$

If only the anisotropic transparent crystal is introduced between the two polarizing filters, the transmission factor of the device D, recorded with a spectrophotometer, is a channeled spectrum characterized by unitary maxima and null minima in the case when the angles between the transmission directions of the polarizing filters and the principal axes of the anisotropic layer are 45°. The positions of the minima and maxima are determined by the phase difference, $\Delta \psi_{1}$, introduced between the ordinary and extraordinary components of light in the propagation time through the anisotropic crystal C.

The method based on channeled spectra has the advantage that the linear birefringence of the anisotropic layers can be estimated in a large spectral range (in the visible range), allowing it to evidence the birefringence dispersion.

When a polymer anisotropic layer is introduced between the crystal C and the analyzer A, the emergent flux becomes the following:

(8) $$\phi_{e}=\frac{\phi_{i}}{2}\left[1\pm \cos\left(\Delta \psi_{1}\pm \Delta \psi_{2}\right)\right]$$

The phase difference, $\Delta \psi_{2}$, introduced by the polymer layer AL between the two components of light depends on both the linear birefringence Δ*n*_2_ and its thickness *L*_2_:

(9) $$\Delta \psi_{2}=\frac{2\pi }{\lambda }\Delta n_{2}L_{2}$$

The supplementary phase difference introduced by the polymer film is added to or subtracted from Δ*ψ*_1_ when the polymer layer is introduced with the stretching direction parallel or perpendicular, respectively, to the crystal optical axis of crystal C. The transmission factor of the device D containing the polymer anisotropic film is written in relation (10):

(10) $$T\left(\lambda \right)=\frac{1}{2}\left[1\pm \cos\left(\Delta \psi_{1}\pm \Delta \psi_{2}\right)\right]$$

It depends on the algebraic sum of the two phase differences, Δ*ψ*_1_ and Δ*ψ*_2,_ introduced by the two anisotropic layers between the ordinary and extraordinary rays.

Let us write the transmission factor of the device D when, between the polarizer and analyzer, both the anisotropic layers C and AL are introduced, with their optical axes in parallel (*p*) or orthogonal to each another (*o*), respectively, taking into consideration that for the orthogonal optical axes, the ordinary ray from crystal plate C becomes extraordinary in the polymer layer and the extraordinary one becomes the ordinary one, as is written in relations (11) and (12):

(11) $$T_{p}\left(\lambda \right)=\frac{1}{2}\left[1\pm \cos\left(\frac{2\pi }{\lambda }\Delta n_{1}L_{1}+\frac{2\pi }{\lambda }\Delta n_{2}L_{2}\right)\right]$$

(12) $$T_{o}\left(\lambda \right)=\frac{1}{2}\left[1\pm \cos\left(\frac{2\pi }{\lambda }\Delta n_{1}L_{1}-\frac{2\pi }{\lambda }\Delta n_{2}L_{2}\right)\right].$$

The sum and the difference of relations (11) and (12) allow the estimation of the linear birefringence of the polymer film:

(13) $$T_{+}\left(\lambda \right)=T_{p}\left(\lambda \right)+T_{o}\left(\lambda \right)$$

(14) $$T_{-}\left(\lambda \right)=T_{p}\left(\lambda \right)-T_{o}\left(\lambda \right)$$

One can express the sum and the difference from (13) and (14) as follows:

(15) $$T_{+}\left(\lambda \right)=1\pm \cos\Delta \psi_{1}\cos\Delta \psi_{2}$$

(16) $$T_{-}\left(\lambda \right)=\mp \sin\Delta \psi_{1}\sin\Delta \psi_{2}.$$

The function from relation (15), $T_{+}\left(\lambda \right)$, written for the crossed polarizing filters, becomes null if $\cos\Delta \psi_{1}=1$ and $\cos\Delta \psi_{2}=1$, and it has maxima for $\cos\Delta \psi_{1}=1$ and $\cos\Delta \psi_{2}=-1$. The function $T_{-}\left(\lambda \right)$ has maxima for the light wavelengths for which $\sin\Delta \psi_{1}=1$ and $\sin\Delta \psi_{2}=-1$ and null values when $\sin\Delta \psi_{2}=0$.

The sum and difference of the experimental spectra offer the possibility to estimate, by their minima and maxima, the linear birefringence of the thin polymer film.

## 4. Results and Discussion

In order to prove the validity of the theory presented in the precedent paragraph, two examples were chosen. For the first time, a PVA film was considered with a known degree of stretching. (The stretching degree was computed as the ratio between the high and small axes of the ellipse in which a circle initially drawn on the film degenerated.)

The channeled spectrum of the thick, transparent, crystalline layer, C, recorded with a spectrophotometer, is given in Figure 3. The values of the linear birefringence of Carpathian quartz correspond to those previously determined [3,4].

**Figure 4:**
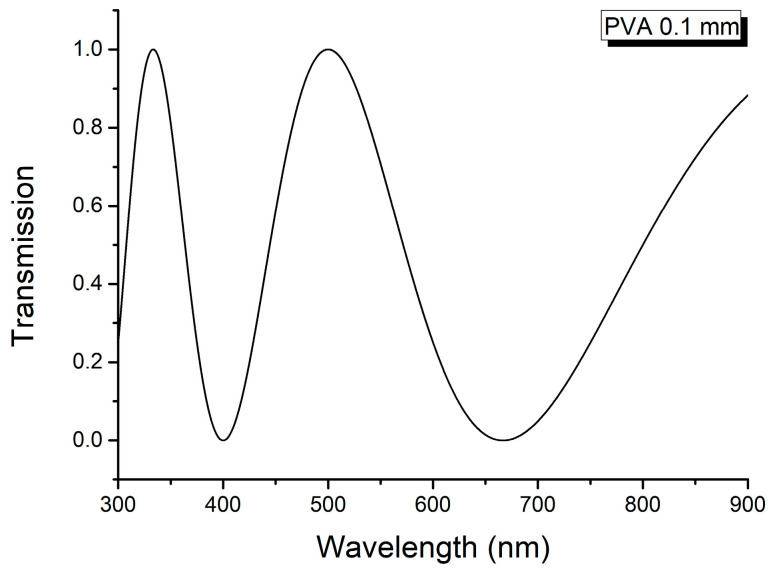
Channeled spectrum of PVA film (*L*_2_ = 10^−1^ mm = 10^−4^ m).

The channeled spectrum of the thin PVA film (see Figure 4) shows only two channels and it is not possible to obtain information about the linear birefringence from this spectrum.

**Figure 5:**
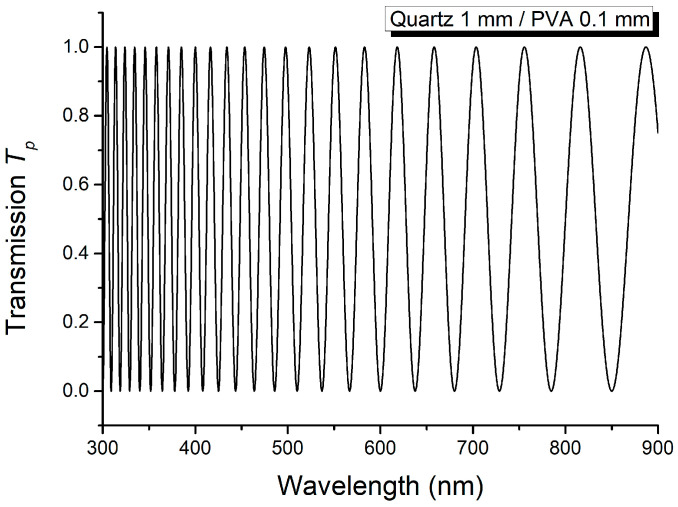
Channeled spectrum for crystalline layer C and PVA polymer film when their optical axes are parallel.

**Figure 6:**
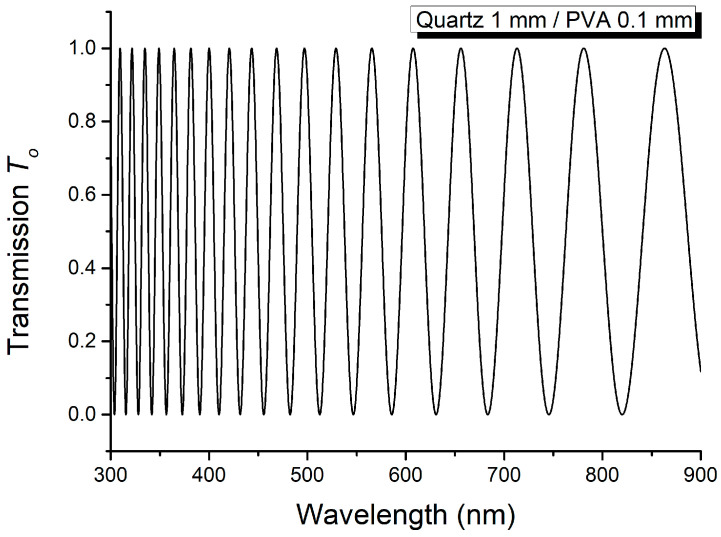
Channeled spectrum for crystalline layer C and PVA polymer film when their optical axes are perpendicular.

The channeled spectrum recorded with the device D containing both crystalline layer C and the thin PVA polymer film, with parallel optical axes, is illustrated in Figure 5, while the same type of spectrum, but for perpendicular optical axes of the two anisotropic layers, is given in Figure 6. The channeled spectrum from Figure 5 shows more channels compared to that from Figure 6, because the phase difference is higher for the first.

**Figure 7:**
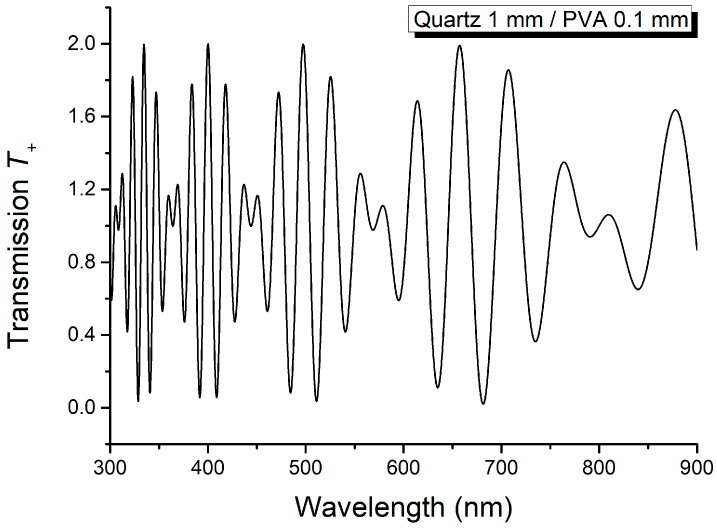
Sum *T*_+_ of the channeled spectra from Figure 5 and Figure 6.

**Figure 8:**
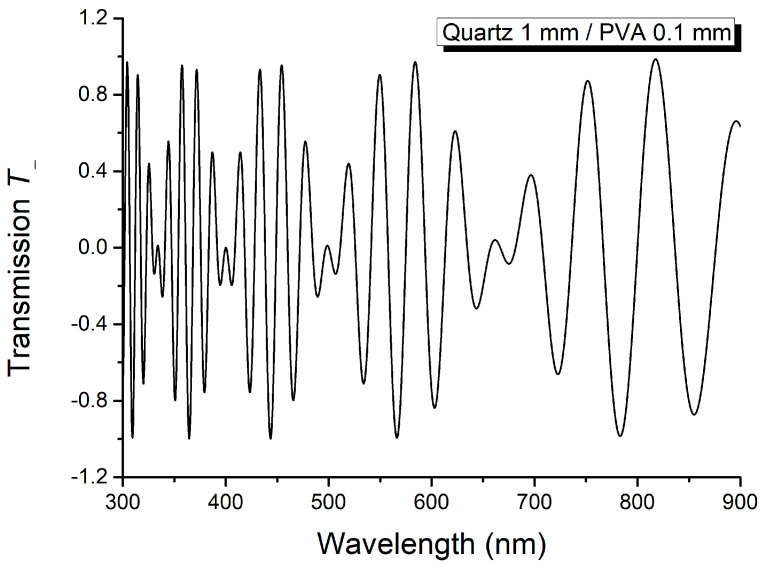
Difference *T*_−_ of the channeled spectra from Figure 5 and Figure 6.

The sum *T*_+_ and difference *T*_−_ of the spectra from Figure 5 and Figure 6 are shown in Figure 7 and Figure 8.

The results obtained using Figure 7 and Figure 8 are given in Table 1 and Table 2. The wavenumbers of the maxima and minima from *T*_+_ and *T*_−_, and the linear birefringence computed according to the theoretical relations, are given in Table 1 and Table 2.

According to relations (15) and (16), the maxima of *T*_+_ are obtained when $\cos\Delta \psi_{2}=-1$, while its null minima correspond to $\cos\Delta \psi_{2}=1$. These conditions are accomplished for Δ*ψ*_2_ = (2*k* + 1)π, *k* = 0, 1, 2, …, and for Δ*ψ*_2_ = 2*k*π, *k* = 0, 1, 2, …, respectively. Function *T*_−_ has its extreme values, +1 and –1, for Δ*ψ*_2_ = (2*k* + 1)π/2, *k* = 0, 2, 4, 6, … and Δ*ψ*_2_ = (2*k* + 1)π/2, *k* = 1, 3, 5, 7, …, respectively.

The linear birefringence of the thin anisotropic layer, listed in Table 1 and Table 2 for the thin film PVA, is computed using the following formula:

(17) $$\Delta n_{2}=\frac{1}{L_{2}}\frac{\lambda_{1}\lambda_{2}}{\lambda_{2}-\lambda_{1}}.$$

The measured values of the wavenumbers in maxima and minima of sum *T*_+_ and difference *T*_−_ are listed in Table 1 and Table 2. The data from Table 1 and Table 2 align with the results obtained in the Theoretical Notions section.

The final result of the measurement of linear birefringence of PVA film is $\Delta n_{2}=0.0203887\pm 0.0000836$.

**Figure 9:**
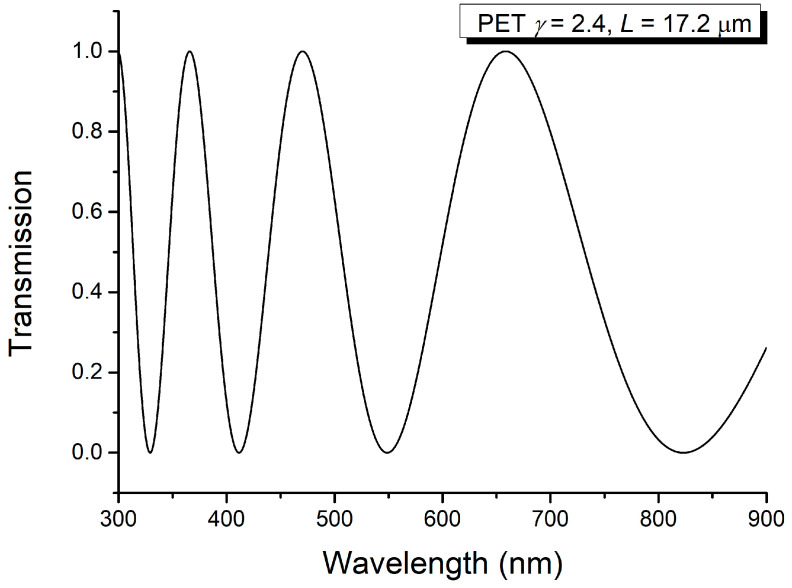
Channeled spectrum of PET film (*L*_2_ = 17.2 μm; *γ* = 2.4).

The applicability of the method proposed in this manuscript is also demonstrated if one considers a thin film of PET for which the values of the linear birefringence are also known [10,11] from other experiments. This is exemplified in Figure 9. Let us illustrate the sum and difference spectra for the thin PET film. The same thick crystalline layer C was used to obtain the channeled spectra of PET thin film.

**Figure 10:**
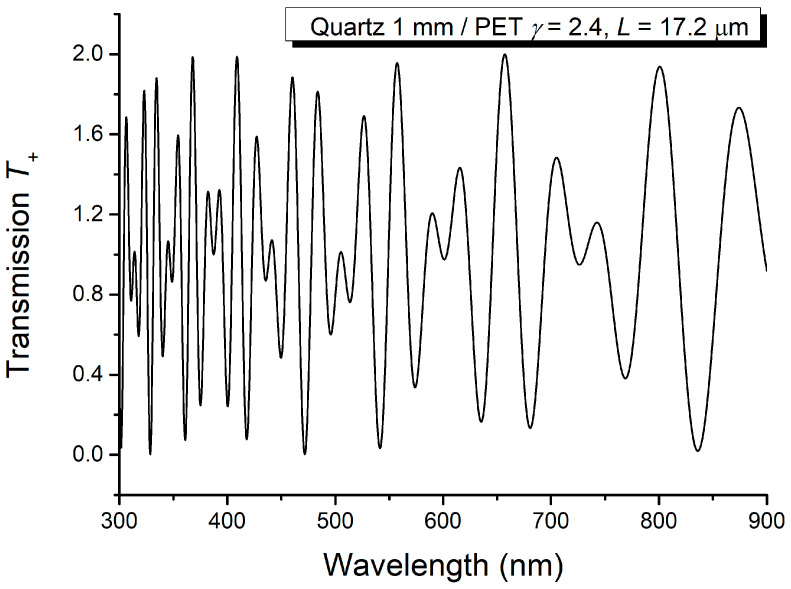
Sum of the channeled spectra *T_o_* and *T_p_* recorded for PET thin film.

**Figure 11:**
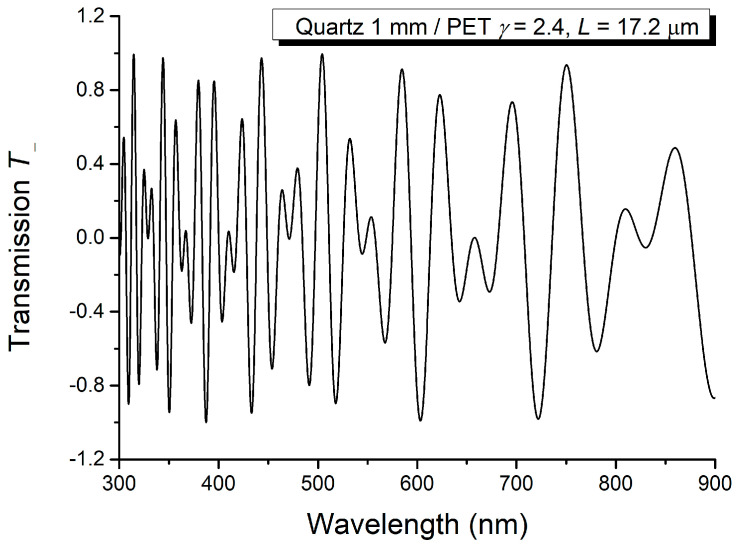
Difference of the channeled spectra *T_p_* and *T_o_* recorded for PET thin film.

Based on the channeled spectra recorded when C and AL have parallel and perpendicular optical axes, respectively, the sum and difference were computed and illustrated in Figure 10 and Figure 11.

The data resulting from the analysis of Figure 10 and Figure 11 are given in Table 3 and Table 4.

The results obtained for the thin PET film correspond with those obtained in [10] for the same degree of stretching.

The final result of the measurement of the linear birefringence of PVA film is $\Delta n_{2}=0.2132335\pm 0.0003214$.

The values of the linear birefringence of the thin PVA and PET films determined by the method described in this article correspond to those determined by other methods for films with the same degree of stretching.

## 5. Conclusions

The rapid and simple method for determining the linear birefringence of thin uniaxial films (polymer or liquid crystals) described in this article can be applied with enough precision for anisotropic materials with high anisotropy.

The interferometric measurements ensure precision for this kind of determination.

The sum and difference of the channeled spectra obtained with two anisotropic layers cut parallel to their optical axes, one with high thickness and the other very thin, used in parallel and perpendicular arrangement of their optical axes allow the determination of the linear birefringence of the thin anisotropic layer, knowing only the positions of the maxima and minima of the sum and/or difference of two channeled spectra in the wavelengths scale.

## Figures and Tables

**Table 1:** Linear birefringence of PVA (*L*_2_ = 0.1 mm; *γ* = 2.40) (P orthogonal to A) computed using the sum of channeled spectra, *T*_+_. $\Delta n_{2}=0.0204185\pm 0.0000168$.

Wavelength (nm)	Δ*n*_2_	Theoretical Relations
Maxima of channeled spectrum		
334.5; 400.0	0.0204275	$T_{+}=1-\cos\Delta \psi_{1}\cos\Delta \psi_{2}$ $\cos\Delta \psi_{2}=-1$ $\bar{\Delta n_{2}}=0.0204337$
400.0; 497.3	0.0204440	
497.3; 657.3	0.0204297	
Minima of channeled spectrum		
328.6; 391.6	0.0204254	$T_{+}=1-\cos\Delta \psi_{1}\cos\Delta \psi_{2}$ $\cos\Delta \psi_{2}=1$ $\bar{\Delta n_{2}}=0.0204033$
408.8; 511.0	0.0204400	
511.0; 682.3	0.0203445	

**Table 2:** Linear birefringence of PVA (*L*_2_ = 0.1 mm; *γ* = 2.40) (P orthogonal to A) computed using the difference of channeled spectra, *T*_−_. $\Delta n_{2}=0.0203589\pm 0.0001505$.

Wavelength (nm)	Δ*n*_2_	Theoretical Relations
Maxima of channeled spectrum		
304.2; 357.2	0.0204362	$T_{-}=-\sin\Delta \psi_{1}\sin\Delta \psi_{2}$ $\sin\Delta \psi_{1}=1$ $,\;\sin\Delta \psi_{2}=-1$ $\bar{\Delta n_{2}}=0.0204352$
454.2; 584.0	0.0204355	
584.0; 817.6	0.0204338	
Minima of channeled spectrum		
309.2; 364.4	0.0204376	$T_{-}=-\sin\Delta \psi_{1}\sin\Delta \psi_{2}$ $\sin\Delta \psi_{1}=1$ $,\;\sin\Delta \psi_{2}=1$ $\bar{\Delta n_{2}}=0.0202827$
364.4; 443.4	0.0204526	
443.4; 566.2	0.0204440	
566.2; 793.2	0.0197968	

**Table 3:** Linear birefringence of PET (*L*_2_ = 17.2 × 10^−6^ m; *γ* = 1.66) (P orthogonal to A) computed using the sum of channeled spectra, *T*_+_. $\Delta n_{2}=0.2133057\pm 0.0002157$.

Wavelength (nm)	Δ*n*_2_	Theoretical Relations
Maxima of channeled spectrum		
367.9; 409.0	0.2131284	$T_{+}=1-\cos\Delta \psi_{1}\cos\Delta \psi_{2}$ $\cos\Delta \psi_{2}=-1$ $\bar{\Delta n_{2}}=0.2130994$
557.3; 657.2	0.2131755	
657.2; 800.7	0.2139944	
Minima of channeled spectrum		
301.6; 328.6	0.2134060	$T_{+}=1-\cos\Delta \psi_{1}\cos\Delta \psi_{2}$ $\cos\Delta \psi_{2}=1$ $\bar{\Delta n_{2}}=0.2133057$
471.7; 541.4	0.2130212	
680.9; 835.9	0.2134900	

**Table 4:** Linear birefringence of PET (*L*_2_ = 17.2 × 10^−6^ m; *γ* = 2.4) (P orthogonal to A) computed using the difference of channeled spectra, *T*_–_. $\Delta n_{2}=0.2131614\pm 0.0004271$.

Wavelength (nm)	Δ*n*_2_	Theoretical Relations
Maxima of channeled spectrum		
314.5; 344.5	0.2131525	$T_{-}=-\sin\Delta \psi_{1}\sin\Delta \psi_{2}$ $\sin\Delta \psi_{1}=1$ $,\;\sin\Delta \psi_{2}=-1$ $\bar{\Delta n_{2}}=0.2130487$
443.2; 504.2	0.2139827	
623.9; 750.4	0.2130108	
Minima of channeled spectrum		
350.2; 387.4	0.2135917	$T_{-}=-\sin\Delta \psi_{1}\sin\Delta \psi_{2}$ $\sin\Delta \psi_{1}=1$ $,\;\sin\Delta \psi_{2}=1$ $\bar{\Delta n_{2}}=0.2132806$
603.1; 721.9	0.2130696	

## Data Availability

The data presented in this study will be available upon request from the corresponding author.
